# Acknowledgement to reviewers of *Cancer Drug Resistance* in 2019

**DOI:** 10.20517/cdr.2020.02

**Published:** 2020-03-19

**Authors:** Cancer Drug Resistance Editorial Office

**Affiliations:** OAE Publishing Inc., Chaoyang District, Beijing 100016, China.

The editors of the journal *Cancer Drug Resistance* (*CDR*) would like to take this opportunity to express their sincere gratitude to the following reviewers for assessing the manuscripts in 2019 [Table 1].

**Table 1:** The reviewers in 2019

Names		
Abdelmohsen, Kotb	Holler, Eggehard	Puri, Sonam
Abe, Tatsuya	Hydbring, Per	Ricciardelli, Carmela
Altucci, Lucia	Jacinto, Estela	Rigaud, Michel
Amantini, Consuelo	Jansen, Gerrit	Ritter, Christoph
Asao, Tetsuhiko	Jenkins, Frank J.	Robert, Jacques
Azmi, Asfar	Jiménez-Morales, Silvia	Roberts, Arthur
Baba, Tomohisa	Jogalekar, Manasi	Rocca, Andrea
Ballinger, James R.	Johns, Terrance	Rodolfo, Monica
Beck, William T.	Jolly, Mohit Kumar	Rodrigues, Antonio Sebastião
Beckermann, Kathryn Eby	Ju, Jingfang	Rodriguez, Jose Antonio
Bednarski, Patrick J.	Kalayda, Ganna	Rodríguez-Antona, Cristina
Benny, Ofra	Kanda, Tatsuo	Rossana, Berardi
Bessho, Tadayoshi	Kannan, Pavitra	Rueff, José
Boland, Genevieve	Kaoud, Tamer	Rupasinghe, H.P. Vasantha
Bottone, Maria Grazia	Ketter, Ralf	Saffi, Jenifer
Bourdon, Jean-Christophe	Keyse, Steven	Sato, Akinori
Branco, Pedro Castelo	Knudsen, Birgitta	Scarpi, Emanuela
Buchbinder, Elizabeth I.	Koczor, Chris	Schwarzenbach, Heidi
Buchler, Tomas	Krajinović, Maja	Shah, Khyati
Budunova, Irina	Kwok, Hang Fai	Shimono, Yohei
Calvert, Hilary	Lahn, Michael M	Simanshu, Dhirendra K.
Cappelletti, Vera	Lauschke, Volker M	Sionov, Ronit Vogt
Carnero, Amancio	Lavrik, Olga	Skaar, Todd C.
Cascorbi, Ingolf	Lee, Yuan-Hao (Chris)	Somasagara, Ranga
Cecchin, Erika	Leo, Eliana	Sordet, Olivier
Cerella, Claudia	Leo, Elisabetta	Sorrentino, Rosalinda
Chen, Fei	Le-Rademacher, Jennifer	Sriuranpong, Virote
Chen, Xiao	Li, Jianfeng	Stein, Ulrike
Christopherson, Richard I	Li, Fengzhi	Sueta, Aiko
Ciccolini, Joseph	Li, Wei	Syed, Nelofer
Curtin, Nicola J.	Li, Xiujun (James)	Szlosarek, Peter W.
Daidone, Maria Grazia	Lo Nigro, Cristiana	Tanaka, Yoichi
Dandawate, Prasad	Mahadevan, Daruka	Terry, Stéphane
Das, Benu Brata	Majello, Barbara	Tripathi, Manish K.
De, Pradip	Manier, Salomon	Tsuda, Naotake
De Lalla, Claudia	Marco, Giuseppetti Gian	Ueda, Takanori
Dellabona, Paolo	Martin, Margarita	Urbatsch, Ina L.
Dharmawardhane, Suranganie	Michaelis, Martin	Vallacchi, Viviana
Domenicotti, Cinzia	Miller, John H.	Van Houten, Bennett E.
Donnini, Sandra	Minderman, Hans	Van Roy, Nadine
Duan, Bin	Mittal, Karuna	Van Tine, Brian A.
Dudás, József	Monica, Silvia La	Van Waardenburg, Robert C.A.M.
Elshimali, Yahya	Morales, Serafin	Venkatachalam, Annapoorna
Essmann, Frank	Morbidelli, Lucia	Verdijk, Robert M.
Falasca, Marco	Morgan, Thomas	Vergara, Daniele
Falconi, Mattia	Morgenstern, Daniel A.	Viola, Giampietro
Fan, Ping	Moro, Loredana	Viswakarma, Navin
Fang, Qingming	Mostaghel, Elahe A.	Vitale, Giovanni
Feun, Lynn G.	Mousa, Shaker	Viveiros, Miguel
Fiandalo, Michael	Mraz, Marek	Wakimoto, Hiroaki
Finlay, Graeme	Muzio, Giuliana	Wei, Yanzhang
Gandhi, Varsha V.	Nakamura, Yuichi	Whittaker, Steven
Gassman, Natalie R.	Neubauer, Hans	Wiemer, Erik A.C.
Gaynon, Paul S	Neuman, Keir	Wilk, Anna
Gemmill, Robert	Ocker, Matthias	Williams, Mark
Ghosh, Chandrayee	Ogino, Shuji	Woolfenden, James M.
Giampieri, Riccardo	Őrfi, László	Wu, Lan-Xiang
Giorgi, Ugo De	Pagano, Michele	Xu, Xiang-Xi
Girotti, Albert	Panchapakesan, Balaji	Yague, Ernesto
Gmeiner, William Henry	Park, Sungsu	Yahya, Elshimali
Golubovskaya, Vita M.	Patil, Tejas	Yamauchi, Takahiro
Grivicich, Ivana	Perry, Jo K.	Yang, Eddy S.
Guo, Xiu-Li	Peterson, Tim	Yee, Douglas
Hadizadeh, Farzin	Pillozzi, Serena	Yoon, Karina
Hanemann, C. Oliver	Pizzimenti, Stefania	Yue, Wei
Hempel, Georg	Pore, Milind	Zatelli, Maria Chiara
Hnízda, Aleš	Pranay, Atul	Zheng, Shu-Sen
Hochmair, Maximilian	Premkumar, Daniel	

We greatly appreciate the contribution they made and the time they donated to the journal. They can get their Reviewer Recognition Certificates by building their peer review profiles either on Publons (https://publons.com) or on ORCID (https://orcid.org/).

If you are interested in becoming a reviewer for *CDR*, you are welcome to apply at https://cdrjournal.com/journal/vol_reviewer.

## Declarations

### Copyright

© The Author(s) 2020.

